# The Role of *Anisakis* sp. in α-Gal Sensitization: Implications for Parasitic-Induced Meat Allergy

**DOI:** 10.3390/pathogens14080789

**Published:** 2025-08-07

**Authors:** Marta Rodero, Sara Romero, Ángela Valcárcel, Juan González-Fernández, A. Sonia Olmeda, Félix Valcárcel, Alvaro Daschner, Carmen Cuéllar

**Affiliations:** 1 Departamento de Microbiología y Parasitología, Facultad de Farmacia, Universidad Complutense de Madrid, 28040 Madrid, Spain; sarahnet96@hotmail.com (S.R.); angelavalcarcelolmeda@gmail.com (Á.V.); juangonzalez@ucm.es (J.G.-F.); 2 UCD Veterinary Sciences Centre, University College Dublin, Belfield, D04 V1W8 Dublin, Ireland; 3 Departamento de Sanidad Animal, Facultad de Veterinaria, Universidad Complutense de Madrid, 28040 Madrid, Spain; angeles@ucm.es; 4 Grupo de Parasitología Animal, Departamento de Reproducción Animal, INIA-CSIC, 28040 Madrid, Spain; valcarcel.felix@inia.csic.es; 5 Instituto de Investigación Sanitaria (IIS)-Servicio de Alergia, Hospital Universitario de La Princesa, 28006 Madrid, Spain; alvarodaschner@gmail.com

**Keywords:** *Anisakis* sp., alpha-Gal epitope, parasitic sensitization, allergic reactions, glycoproteins, cross-reactivity

## Abstract

**Background/Objectives:** This study investigates the potential of *Anisakis* sp. as a novel source of α-Gal (Galα1-3Galβ1-4GlcNAc-R) epitopes capable of inducing allergic sensitization in humans. While α-Gal is classically associated with delayed IgE-mediated hypersensitivity following tick bites, emerging evidence suggests that parasitic helminths such as *Anisakis* sp. may also express α-Gal-containing glycoconjugates, offering an alternative sensitization pathway. **Methods:** Protein extracts from *Anisakis* sp. third-stage larvae and mammalian tissues (beef, pork) were analyzed by SDS-PAGE and Western blot using a monoclonal anti-α-Gal antibody (clone M86), and α-Gal epitopes were detected by ELISA. Sera from urticaria patients, stratified by *Anisakis* sp. sensitization status, were evaluated for anti-α-Gal IgG, IgE, and IgG4 antibodies. Inhibition assays assessed cross-reactivity. **Results:** Results confirmed the presence of α-Gal epitopes on *Anisakis* sp. proteins, with prominent bands at ~250 kDa and 65 kDa. Urticaria patients sensitized to *Anisakis* sp. exhibited significantly elevated anti-α-Gal antibody levels compared to controls. Inhibition ELISA demonstrated substantial reduction in antibody binding with *Anisakis* sp. extracts, indicating shared antigenic determinants with mammalian α-Gal. **Conclusions:** These findings establish *Anisakis* sp. as a source of α-Gal-containing glycoproteins capable of eliciting specific antibody responses in humans, highlighting a potential parasitic route for α-Gal sensitization.

## 1. Introduction

The α-Gal epitope (Galα1-3Galβ1-4GlcNAc-R) is widely expressed on glycolipids and glycoproteins of non-primate mammals and New World monkeys, but absent in humans, apes, and Old World monkeys due to inactivation of the GGTA1 gene encoding α1,3-galactosyltransferase [1,2,3]. This evolutionary loss, occurring 20–30 million years ago, led to the development of natural anti-α-Gal antibodies in these species. In humans, high levels of anti-α-Gal antibodies are produced in response to exogenous α-Gal from microbes, impacting immune processes such as xenotransplant rejection and pathogen neutralization [4]. Sensitization to the highly immunogenic α-Gal epitope in mammalian tissues can induce an IgE-mediated immune response in some individuals, resulting in α-Gal syndrome (AGS) or red meat allergy. This condition manifests as an allergic reaction following the ingestion of mammalian meat containing the α-Gal epitope [5].

AGS presents with IgE-mediated hypersensitivity reactions, including urticaria, pruritus (notably on palms, soles, and ears), and recurrent angioedema [6,7]. Around 60% of patients report gastrointestinal symptoms such as abdominal pain, diarrhoea, nausea, and vomiting, with up to 20% experiencing isolated gastrointestinal manifestations resembling functional bowel disorders [7]. Severe AGS cases can progress to anaphylaxis, characterized by hypotension, respiratory distress, and cardiovascular instability, typically 3–8 h after ingesting α-Gal-containing foods due to delayed glycolipid absorption. Reaction severity correlates with α-Gal epitope density, with organ meats and game animals posing a higher risk than processed meats or dairy. Cofactors such as alcohol, NSAIDs, and physical exertion may lower reaction thresholds by influencing immune or metabolic pathways, though their precise mechanisms remain under investigation [7,8,9,10].

Diagnosis of AGS relies on detecting α-Gal-specific IgE (>0.1 IU/mL), with variable symptom onset and organ involvement [7]. Some patients develop tolerance over time, indicating dynamic immune regulation [6,9]. Substantial evidence implicates tick bites, especially from Ixodidae species, as critical in sensitizing individuals to α-Gal epitopes and initiating AGS [11,12]. Sensitization occurs via the introduction of α-Gal glycoproteins or glycolipids present in tick saliva during feeding [11,12,13]. Studies have shown that ticks such as *Amblyomma sculptum* and *Ixodes ricinus* possess salivary α-Gal epitopes capable of inducing anti-α-Gal IgE and IgG responses, particularly in α-1,3-galactosyltransferase knockout models. These responses are Th2-polarized, promoting α-Gal-specific IgE production [11,12,13]. Epidemiological and experimental data consistently link repeated tick exposures to increased α-Gal-specific IgE titres and heightened risk of mammalian meat hypersensitivity [8,11,12]. Immunologically, both direct exposure to tick-derived α-Gal antigens and saliva-mediated immune modulation, and suppression of pro-inflammatory cytokines and enhancement of IL-10 and TGF-β, contribute to this process [8,11,12].

The first documented AGS case occurred in Australia in 2007, with subsequent cases reported in 17 countries [8]. In Spain, incidence rates show significant geographic variation, with higher prevalence in rural northern regions compared to other areas [14,15,16].

*Anisakis* sp., a parasitic nematode of the family Anisakidae, is the etiological agent of anisakiosis. Infection occurs after ingesting live third-stage larvae in raw or undercooked marine fish or cephalopods, which penetrate the gastrointestinal mucosa [17]. Anisakiosis manifests through both mechanical tissue damage and immunological sensitization, paralleling allergic gastroenteritis. Larval proteolytic enzymes enable mucosal invasion and induce a Th2 immune response with eosinophil infiltration and increased IL-4, IL-5, and IL-13 production [18]. This cascade drives parasite-specific IgE synthesis, predisposing to hypersensitivity reactions such as urticaria, angioedema, and anaphylaxis [18,19].

*Anisakis* sp. infection involves both invasive and allergic mechanisms: acute symptoms such as abdominal pain, nausea, and vomiting arise soon after larval ingestion, while chronic forms may mimic inflammatory bowel diseases due to granulomatous responses [20]. Sensitization persists beyond larval removal, as allergenic proteins from excretory/secretory products sustain immunological memory. This overlap between parasitic invasion and IgE-mediated hypersensitivity highlights the complex interplay of infectious and allergic processes in anisakiosis [18,19,20,21].

Several studies indicate that helminth infections, particularly *Ascaris lumbricoides*, may contribute to α-Gal sensitization [22], while infection with *Toxocara canis* inhibits the production of IgE antibodies to α-Gal [23]. Elevated anti-α-Gal IgE levels in helminth-endemic regions [22] suggest potential synergistic interactions between parasitic exposures and environmental cofactors in driving allergic sensitization pathways.

This investigation aims to characterize the presence of α-Gal epitopes within the protein crude extract of *Anisakis* sp. and evaluate their potential role in sensitization pathways. The study will analyze serum samples from urticaria patients, stratified by *Anisakis* sp. sensitization status, to detect specific anti-α-Gal antibodies. We hypothesize that *Anisakis* sp. may express α-Gal-containing glycoproteins or glycolipids capable of inducing IgE-mediated sensitization to this carbohydrate epitope. This work addresses a critical gap in understanding carbohydrate-mediated sensitization in parasitic infections and its intersection with food allergy pathogenesis. The findings may clarify whether *Anisakis* sp. exposure represents a novel route for α-Gal sensitization, potentially explaining atypical urticaria phenotypes in endemic regions.

## 2. Materials and Methods

### 2.1. Protein Crude Extract Preparation

To prepare protein crude extracts from diverse biological samples, skeletal muscle tissues from beef (*Bos taurus*) and pork (*Sus scrofa domesticus*) were obtained from commercial sources, while third-stage larvae (L3) of *Anisakis* sp. were manually isolated from the visceral organs, musculature, and coelomic cavities of naturally infected blue whiting (*Micromesistius poutassou*) (FAO area 27), followed by extensive washing in deionized water to remove host residues [24].

All biological specimens underwent mechanical disruption using a chilled glass tissue homogenizer (4 °C) in phosphate-buffered saline (PBS; 0.01 M phosphate, 0.137 M NaCl, pH 7.4) to maintain protein stability. The homogenates were subjected to ice-cold extraction for 10 min to facilitate protein solubilization. Crude extracts were clarified by centrifugation at 6700× *g* for 10 min (4 °C) to separate insoluble cellular debris from the soluble protein fraction. The supernatant containing the soluble proteome was collected for subsequent analyses.

For *Anisakis* sp. larvae, delipidated antigens were generated using established protocols involving organic solvent extraction to remove lipid components while preserving protein immunogenicity. The homogenate was extracted in PBS at 4 °C overnight, delipidized with n-hexane, and centrifuged for 30 min at 4 °C. This method enhances antigen specificity for immunological applications by eliminating non-proteinaceous interferents [25].

### 2.2. Serum Samples

The study population was prospectively enrolled during the same recruitment period from a single geographic location (Madrid, Spain), adhering to the diagnostic criteria established by González-Fernández et al. in 2024. Participants were stratified into three cohorts: gastro-allergic anisakiosis (GAA) patients (n = 13), chronic urticaria with *Anisakis* sp. sensitization (CU+) (n = 18), and chronic urticaria without *Anisakis* sp. sensitization (CU−) (n = 23).

All participants provided written informed consent prior to enrolment. The study protocol received ethical approval from the Institutional Review Board of the University Hospital La Princesa, Madrid (Protocol No. PI-515-07/04/11). The samples had been utilized in several previous studies [26,27].

### 2.3. Detection of α-Gal Epitopes in Anisakis sp. Crude Protein Extract via Indirect ELISA

The presence of proteins bearing α-Gal epitopes in the non-delipidated extract of *Anisakis* sp. was analyzed using an indirect enzyme-linked immunosorbent assay (ELISA) methodology. Nunc MaxiSorp™ (Thermo Fisher Scientific, Carlsbad, CA, USA) plates were coated with 10 μg/mL of extract diluted in PBS, followed by blocking of residual binding sites to minimize nonspecific interactions. A monoclonal mouse anti-α-Gal antibody (clone M86, Enzo Life Sciences, Farmingdale, NY, USA), specific for the Galα1-3Galβ1-4GlcNAc-R epitope, was applied at a dilution of 1:4 and incubated overnight at 4 °C. Subsequently, horseradish peroxidase (HRP)-conjugated rabbit anti-mouse IgM secondary antibody (Catalog #31456, Invitrogen, Thermo Fisher Scientific, Carlsbad, CA, USA) was introduced at a 1:500 dilution, with incubation for 1 h at 37 °C. Colorimetric detection was achieved using o-phenylenediamine (OPD; Sigma-Aldrich, Bayern, Germany) as the chromogenic substrate in the presence of hydrogen peroxide, with the enzymatic reaction terminated by addition of 3 M sulfuric acid. Absorbance values were quantified spectrophotometrically at 490 nm [28].

This protocol leverages the high specificity of the M86 monoclonal antibody for α-Gal-containing glycoconjugates, ensuring minimal cross-reactivity with unrelated carbohydrate structures. The IgM isotype of the primary antibody, combined with stringent blocking and dilution conditions, optimizes sensitivity for low-abundance epitopes while maintaining assay reproducibility.

### 2.4. Polyacrylamide Gel Electrophoresis (SDS-PAGE)

Electrophoresis was performed using a Mini-PROTEAN^®^ system (Bio-Rad, Hercules, CA, USA), comprising a 4% stacking gel and a 10% resolving gel.

Non-delipidated extract (1 mg) from *Anisakis* sp. was diluted in denaturing sample buffer (50 mM Tris-HCl, pH 8.6, 2% SDS, 20% glycerol, 0.02% bromophenol blue) and mixed 1:1 with electrophoresis buffer (25 mM Tris, 192 mM glycine, 1% SDS, pH 8.3). The mixture was heat-denatured prior to loading.

Samples were resolved at a constant voltage of 100 V for 2 h in Tris-glycine-SDS running buffer (25 mM Tris, 192 mM glycine, 0.1% SDS). Precision Plus Protein Kaleidoscope^®^ molecular weight markers (10–250 kDa; Bio-Rad) were included for size calibration.

### 2.5. Western Blot

Following SDS-PAGE, proteins were electrophoretically transferred to 0.22 μm nitrocellulose membranes (Pharmacia, Stockholm, Sweden) using a Mini Trans-Blot system (Bio-Rad) at 100 V for 1.5 h in transfer buffer (25 mM Tris, 192 mM glycine, 20% *v*/*v* methanol, pH 8.3). Membranes were sectioned into 20 strips, each representing 50 μg of protein, and blocked overnight at 4 °C with 5% non-fat milk in phosphate-buffered saline (PBS).

Immunodetection was performed by incubating strips with mouse anti-α-Gal antibody (Galα1-3Galβ1-4GlcNAc-R monoclonal antibody M86, Enzo Life Sciences) diluted 1:4 in PBS containing 0.1% Tween 20 (PTB) at 4 °C overnight. Membranes were washed five times (5 min each) with PBS-Tween and probed with horseradish peroxidase (HRP)-conjugated rabbit anti-mouse IgM (Invitrogen, ThermoFisher Scientific; Catalog #31456) at 1:250 dilution for 2 h at room temperature.

Protein bands were visualized using 3,3′-diaminobenzidine tetrahydrochloride (DAB) substrate in PBS with 0.01% hydrogen peroxide. The reaction was terminated by extensive washing with distilled water.

### 2.6. Measurement of Anti-Anisakis sp.-Specific Antibody Levels by ELISA

Specific antibody levels were quantified using a standardized enzyme-linked immunosorbent assay (ELISA). Briefly, 96-well microtiter plates (Costar, Corning, NY, USA) were coated overnight at 4 °C with 10 μg/mL of either non-delipidated (*Anisakis*, beef and pork tissues) or delipidated (*Anisakis*) extracts diluted in carbonate buffer (0.1 M, pH 9.6). Following coating, wells were blocked with an appropriate buffer to minimize nonspecific binding. Human serum samples were diluted 1:100 in PBS-Tween containing 0.1% bovine serum albumin (BSA) and incubated in duplicate at 37 °C for 2 h.

For detection, horseradish peroxidase (HRP)-conjugated goat anti-human secondary antibodies (specific for IgM, IgG, IgA, or IgG4; Biosource International, Camarillo, CA, USA) were applied at optimized dilutions and incubated for 1 h at 37 °C [27,29,30]. The enzymatic reaction was initiated using o-phenylenediamine (OPD; Sigma-Aldrich, Germany) substrate supplemented with hydrogen peroxide and terminated by adding 3 M sulfuric acid. Absorbance was measured at 490 nm using a microplate reader.

For the IgE determination test, sera were added at 1:2 dilution. A murine monoclonal antibody (mAb) against an epsilon human IgE chain (IgG1κ, E21A11, INGENASA, Madrid, Spain) was added, followed by a HRP-conjugated goat anti-mouse IgG1 (gamma) (Life Technologies, Grand Island, NY, USA) [27].

### 2.7. Anti-Alpha Gal-Specific IgM, IgG, IgG4, IgA, and IgE Antibodies

PolySorp plates (Costar, Corning, NY, USA) were pre-treated overnight at 4 °C with 200 µL/well of 2% glutaraldehyde in PBS, pH 5. The plates were washed with PBS (pH 5) and then sensitized overnight with 100 µL/well of 1 μg/mL of epitope Galα1-3Gal-BSA (3-atom spacer, product code: NGP0203, Dextra, UK). For IgG and IgM detection, plates were directly sensitized with 0.5 μg/mL of epitope. Wells were blocked, and patient sera were incubated overnight at 1:100 dilution in duplicate. HRP-conjugated anti-human antibodies, as well as IgE determination, followed the previously described steps.

### 2.8. Inhibition ELISA

A mixture of ten anti-*Anisakis* IgG/IgE-positive sera (SAK) and a separate mixture of ten anti-α Gal IgG/IgE-positive sera (SAG) were diluted 1:200 in 5% bovine serum albumin (BSA) and subjected to overnight adsorption with *Anisakis* sp. non-delipidated extract at a ratio of 600 µL serum per 200 µg protein. Following centrifugation at 6700× *g* for 15 min, supernatants were collected.

Nunc MaxiSorp™ ELISA plates were coated overnight with 100 µL/well of either 10 µg/mL Galα1-3Gal-BSA epitope (Dextra, UK) or *Anisakis* sp. non-delipidated extract. Adsorbed and non-adsorbed SAK and SAG samples were incubated in duplicate at 37 °C for 2 h. Anti-human IgG horseradish peroxidase (HRP) conjugate detection was performed as previously described.

The percentage inhibition was calculated using the following formula:

Inhibition (%) = [O.D. sera non adsorbed − O.D. sera adsorbed/O.D. sera non adsorbed] × 100

### 2.9. Data Analysis

The quantitative variables were analyzed using parametric or non-parametric tests based on distributional assumptions. Normality was assessed through the Kolmogorov–Smirnov test, with a significance threshold of *p* < 0.05. Student’s *t*-test was applied for group comparisons when data adhered to normality assumptions, while the Mann–Whitney *U* test was employed for non-normally distributed datasets. Statistical significance was defined at *p* < 0.05. All analyses were conducted using GraphPad Prism version 6.0 (GraphPad Software, San Diego, CA, USA).

## 3. Results

### 3.1. Detection of Proteins with α-Gal Epitopes in the Non-Delipidated Extract of Anisakis sp.

**Figure 1:**
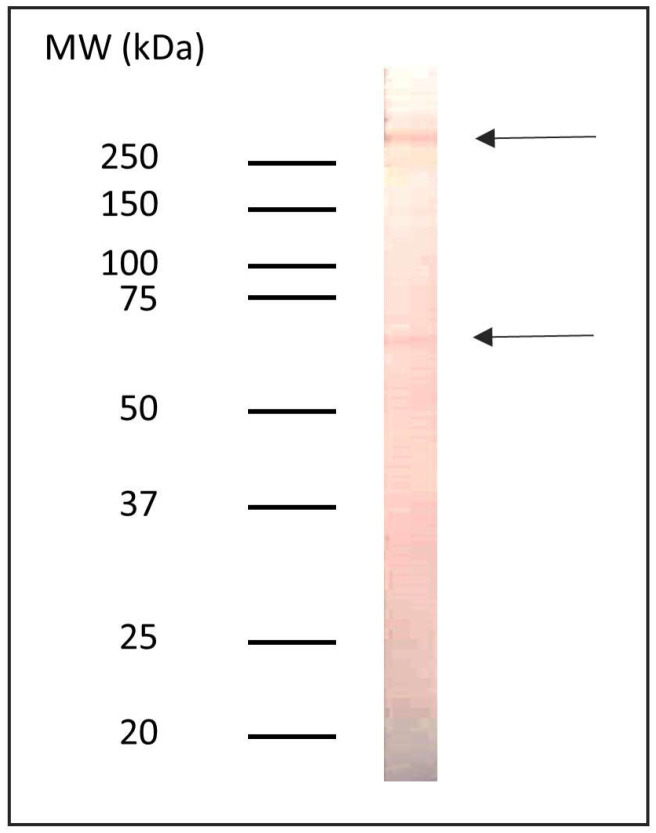
Immunoreactivity profile of proteins containing α-Gal epitopes in the *Anisakis* sp. delipidated protein extract.

The presence of proteins containing α-Gal epitopes in the non-delipidated extract of *Anisakis* sp. was detected by indirect ELISA using a monoclonal anti-α-Gal antibody. The measured values were nearly six times higher than those of the negative control (0.851 ± 0.006 vs. 0.156 ± 0.003). Western blot analysis further confirmed that the reactive proteins had molecular masses of approximately 250 kDa and 65 kDa (Figure 1).

### 3.2. Anti-Anisakis sp. Specific Antibodies

Upon analysis of patient sera with the delipidated *Anisakis* sp. antigen, the GAA group consistently exhibited the highest levels of specific antibodies, with IgG being the predominant isotype detected. Statistically significant differences in antibody levels were observed between *Anisakis*-positive (GAA and CU+) and *Anisakis*-negative (CU−) groups for all immunoglobulin isotypes except IgM. Importantly, the absence of chronic urticaria (GAA group) was associated with significantly higher levels of specific IgG, IgE, and IgG4 compared to groups with urticaria (CU+ and CU−) (Figure S1).

Comparable patterns were observed when sera were tested with the *Anisakis* sp. non-delipidated extract. Elevated levels of specific IgG, IgE, and IgG4, along with a marked decrease in IgA levels, were detected. Again, the absence of chronic urticaria was significantly associated with increased levels of these immunoglobulins (Figure S2A–E).

Analysis of the specific IgE/IgG4 ratio revealed significant differences only between the GAA and CU− groups when tested with the *Anisakis* sp. crude protein extract, suggesting that this ratio may serve as a potential biomarker for distinguishing *Anisakis*-positive from *Anisakis*-negative patients (Figure S2F).

### 3.3. Anti-α-Gal Specific Antibodies

**Figure 2:**
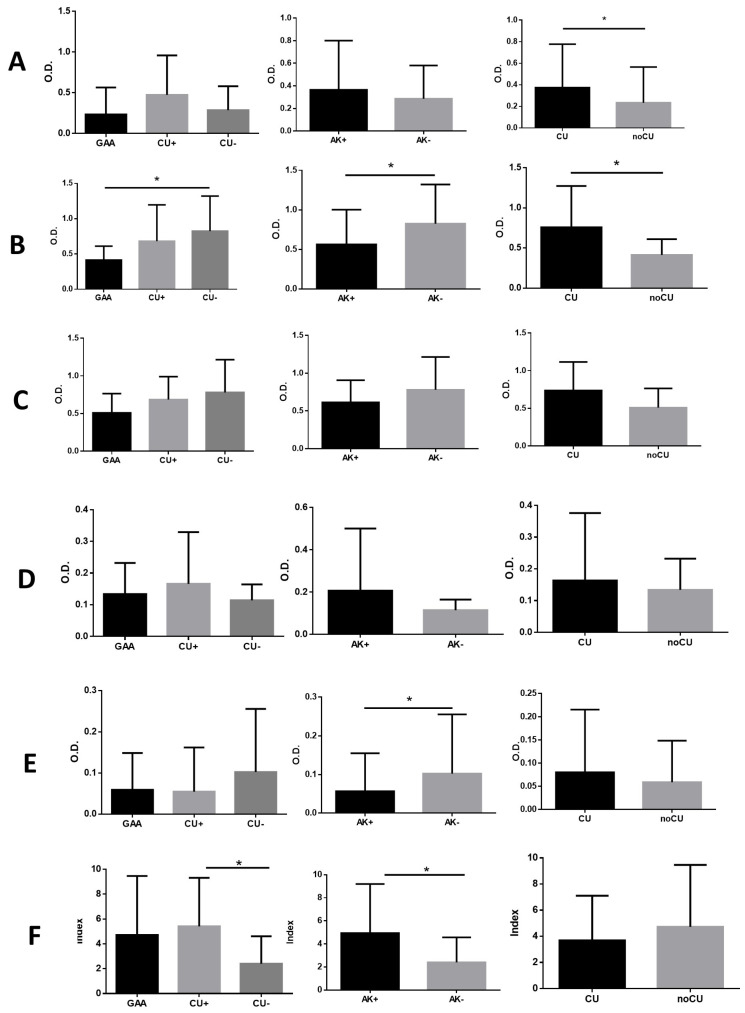
Levels of anti-α-Gal IgG (**A**), IgM (**B**), IgA (**C**), IgE (**D**), IgG4 (**E**), and IgE/IgG4 ratio (**F**) antibodies, expressed as optical densities (O.D.), in patients diagnosed with gastroallergic anisakiosis (GAA), chronic urticaria with sensitization to *Anisakis* sp. (CU+), and chronic urticaria without sensitization to *Anisakis* sp. (CU−). AK+: GAA and CU+ patients; AK−: CU− patients; CU: CU+ and CU− patients; non-CU: GAA patients. Group comparisons were performed using Student’s *t*-test for normally distributed data and the Mann–Whitney *U* test for non-normally distributed data. * *p* < 0.05.

The most pronounced immune response against the α-Gal epitope was observed at the IgM level, with the non-sensitized group (CU−) exhibiting significantly higher specific IgM levels compared to *Anisakis*-positive patients (GAA and CU+) (Figure 2B).

Patients with chronic urticaria (CU+ and CU−) consistently demonstrated higher antibody levels than those without chronic urticaria (GAA), with statistically significant differences observed for both specific IgG and IgM levels (Figure 2A,B).

In contrast, *Anisakis*-positive patients (GAA/CU+) exhibited lower levels of anti-α-Gal IgG4, with significant differences compared to the *Anisakis*-negative group (CU−) (Figure 2E).

### 3.4. Detection of IgG, IgE, and IgG4 Antibodies Against Proteins Present in Mammalian Muscle Tissue

The presence of antibodies against water-soluble protein extracts from beef and pork muscle tissue was evaluated in sera from patients diagnosed with GAA, CU+, and CU−. The highest antibody levels were observed for IgG, with consistently stronger responses to beef compared to pork. However, no statistically significant differences were detected among the different patient groups with respect to *Anisakis* sp. sensitization status or the presence or absence of chronic urticaria (Figure S3A).

IgG4 levels exhibited a pattern similar to that of IgG, with a greater response to beef than to pork. Nonetheless, no significant differences were found among the clinical groups, *Anisakis* sp. sensitization status, or chronic urticaria status (Figure S3B).

In contrast, IgE levels were consistently higher in response to pork than to beef, although these differences were not statistically significant across the studied groups. Slightly elevated IgE levels were observed in the non-sensitized group (CU-), while the presence or absence of chronic urticaria did not significantly influence IgE levels (Figure S3C).

### 3.5. Inhibition ELISA for the Detection of Anti-Anisakis sp. and Anti-α-Gal Antibodies in Serum Samples

IgG levels against the α-Gal epitope and *Anisakis* sp. antigen were evaluated using inhibition ELISA in two pooled serum samples: one consisting of 10 patients sensitized to *Anisakis* (SAK), and another consisting of 10 patients with high IgE levels against the α-Gal epitope (SAG).

**Figure 3:**
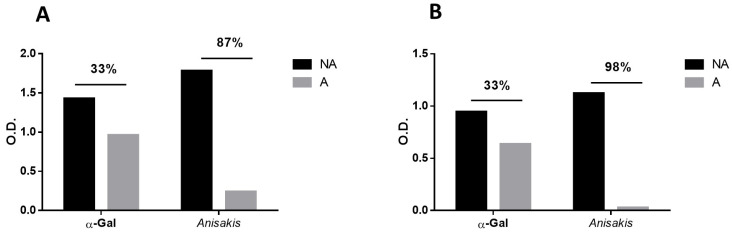
Anti-α-Gal and anti-*Anisakis* sp. non-delipidated IgG levels, expressed as optical densities (O.D.), in pooled SAG (**A**) and SAK (**B**) serum samples before (NA: non-adsorbed) and after adsorption (A) with 200 µg of *Anisakis* sp. non-delipidated antigen. The percentage of inhibition is indicated.

After absorption with 200 µg of *Anisakis* sp. antigen, both the SAK and SAG serum pools exhibited a 33% reduction in anti-α-Gal IgG levels when tested against the α-Gal epitope. *A similar* inhibitory effect was observed for anti-*Anisakis* sp. IgG levels, with an 87% reduction in the SAK group and a near-complete inhibition (98%) in the SAG group (Figure 3).

## 4. Discussion

The α-Gal epitope (Galα1, 3Galα1, 4GlcNAc-R), constitutively expressed in non-primate mammals but absent in humans and Old World primates, triggers protective anti-α-Gal IgM/IgG responses against pathogens (e.g., bacteria, parasites) [1,2,3,4]. However, sensitized individuals produce IgE antibodies against α-Gal, leading to delayed type I allergic reactions (urticaria, anaphylaxis) upon consuming red meat [6]. This sensitization is strongly linked to tick bites, which introduce α-Gal-containing salivary antigens [13,28,31,32]. Additionally, helminths express α-Gal epitopes, correlating with elevated anti-α-Gal antibodies in endemic populations with chronic parasitic infections [5,33]. This study investigates whether larval products of *Anisakis* sp. contain α-Gal-expressing proteins capable of inducing anti-α-Gal antibodies, potentially sensitizing individuals and triggering allergic reactions post-red meat consumption. By identifying α-Gal in *Anisakis*, the research aims to elucidate a novel parasitic pathway for α-Gal sensitization, expanding understanding of cross-reactive allergies beyond tick-mediated mechanisms. Findings could inform clinical management of meat allergies in regions with high *Anisakis* exposure or co-endemicity with helminth infections.

To date, the presence of α-Gal epitopes on proteins from *Anisakis* sp. L3 larvae had not been described. However, other helminths can produce α-Gal, and protein glycosylation is thought to enhance immunogenicity and Th2 responses, as observed in the parasitic helminth *Schistosoma mansoni* [22,34,35,36].

Our study provides new insights into the immunological interplay between *Anisakis* sp. infection and sensitization to the α-Gal epitope, particularly in patients with urticaria. This study is the first to demonstrate the presence of proteins containing α-Gal epitopes in the non-delipidated crude extract of *Anisakis* sp., as confirmed by both indirect ELISA and Western blot analysis. The detection of these epitopes suggests that *Anisakis* sp. may serve as a previously unrecognized source of α-Gal exposure in humans, with potential implications for the development of anti-α-Gal antibody responses. On one hand, antigen exposure may occur through contact with fish containing dead *Anisakis* larvae, particularly in the CU+ group. On the other hand, exposure to a factor released by live larvae that induces immunoglobulin class switching may be involved, especially in GAA patients.

Consistent with previous reports, the highest levels of anti-α-Gal antibodies in human sera were observed at the IgM isotype, reflecting the natural occurrence of these antibodies in individuals lacking endogenous α-Gal due to evolutionary inactivation of the GGTA1 gene [37]. Notably, patients with *Anisakis* sensitization exhibited altered anti-α-Gal antibody profiles compared to non-sensitized individuals, with significant reductions in IgM and IgG4 levels. These findings suggest that *Anisakis* sp. infection may modulate the anti-α-Gal immune response, potentially through antigenic stimulation or immune regulation mechanisms.

The immunoreactivity patterns observed in patients with chronic urticaria further support a link between *Anisakis* infection, α-Gal sensitization, and allergic manifestations. Patients with chronic urticaria and *Anisakis* sensitization (CU+) exhibited higher levels of anti-*Anisakis* and anti-α-Gal IgG and IgM antibodies than those without chronic urticaria or sensitization, indicating a heightened humoral response in this subgroup. However, the absence of chronic urticaria (GAA group) was associated with increased levels of IgG, IgE, and IgG4, suggesting that clinical phenotype may influence the nature of the antibody response.

High levels of anti-α-Gal IgG are found in immunocompetent humans, who cannot synthesize this epitope. These natural antibodies arise from continuous stimulation by microbiota [1]. Upon exposure to foreign α-Gal antigens, B cells are activated, producing high-affinity anti-α-Gal antibodies [4].

However, anti-α-Gal IgG levels may be reduced in individuals with blood group B, as the B antigen structurally resembles the α-Gal epitope, potentially conferring protection against α-Gal syndrome [1]. Our study lacks blood group data, but investigating whether lower anti-α-Gal IgG levels correlate with blood group B could provide insights into possible self-tolerance mechanisms [38].

Alpha-Gal syndrome requires specific IgE production against the α-Gal epitope [5,6,39]. In this study, CU− patients exhibited the lowest anti-α-Gal IgE levels, while GAA and CU+ groups showed comparable values. *Anisakis*-positive patients (GAA/CU+) demonstrated twofold higher anti-α-Gal IgE levels than negatives. This elevation likely stems from prior exposure to α-Gal epitopes released by L3 larvae antigens in raw/undercooked fish consumption. It is also possible, as previously mentioned, that a factor present in the excretory/secretory products of live larvae may induce immunoglobulin class switching toward IgE production. Cabezas-Cruz et al. previously proposed that tick salivary prostaglandin E2 triggers antibody class switching in mature B cells, leading to increased levels of anti-α-Gal IgE antibodies [40].

Anti-α-Gal IgE positively correlates with *Ascaris lumbricoides* IgE in α-Gal allergy, suggesting GAA and CU+ patients’ susceptibility to α-Gal syndrome [21].

Exposure to the α-Gal epitope alone does not induce an IgE response without prior sensitization, typically associated with elevated anti-α-Gal IgG levels, as seen after tick bites [41,42]. In our study, patients, especially those *Anisakis*-positive, exhibited high anti-α-Gal IgG levels. This suggests that sensitization may also occur via contact with *Anisakis* L3 larvae, whose proteins contain α-Gal epitopes, potentially promoting IgG-to-IgE class switching in susceptible individuals and triggering α-Gal syndrome.

Interestingly, inhibition ELISA experiments revealed that absorption with *Anisakis* sp. antigen substantially reduced anti-α-Gal IgG levels in pooled sera from both *Anisakis*-sensitized and α-Gal-sensitized patients, indicating partial antigenic cross-reactivity between *Anisakis* sp. proteins and the α-Gal epitope. This observation raises the possibility that exposure to *Anisakis* sp. may contribute to α-Gal sensitization in certain individuals, potentially explaining the occurrence of hypersensitivity reactions to mammalian meat in regions with high prevalence of anisakiosis [43].

The detection of anti-mammalian muscle tissue antibodies (beef and pork) in all patient groups, regardless of *Anisakis* sensitization or chronic urticaria status, suggests that cross-reactive carbohydrate determinants, such as α-Gal, may be widely recognized in the studied population. Nevertheless, the lack of significant differences among clinical groups indicates that additional factors, including genetic predisposition, environmental exposures, or co-infections, likely modulate the clinical expression of α-Gal-related hypersensitivity [6].

This study presents several limitations that warrant consideration. The restricted sample size and recruitment from a single geographic region may constrain statistical power and limit the generalizability of findings. Crucially, key cofactors including dietary habits, tick bite history, environmental exposures, viral infection status, microbiome composition, and age-related immunological variations were not systematically assessed. This omission introduces potential confounding variables that could obscure the interpretation of α-Gal sensitization pathways.

Additionally, while α-Gal epitopes were identified in *Anisakis* sp. extracts, the specific glycoproteins or glycolipids responsible for sensitization remain uncharacterized, impeding mechanistic understanding. Future investigations will address these gaps through expanded multi-center cohorts, comprehensive profiling of all noted cofactors (including viral, microbiome, and age variables), and integrated proteomic/glycomic analyses to elucidate sensitization mechanisms.

In this study, antibody detection was performed using indirect ELISA, thus primarily identifying immunoglobulins against glycoproteins. However, glycolipids sharing the α-Gal epitope can also induce humoral responses and have been implicated in red meat [44], peach [45], and cow’s milk [46] allergies. Assessing antibodies against glycolipids in patient sera is warranted, as these may be more abundant and relevant, particularly regarding *Anisakis* sp. antigens. Notably, IgE antibodies targeting α-Gal on proteins can also trigger allergic reactions to the same epitope on glycolipids, highlighting their potential role in mammalian meat allergy development [47].

## 5. Conclusions

This work identifies *Anisakis* sp. as a potential contributor to α-Gal sensitization in humans and underscores the need for heightened awareness of parasitic infections as risk factors for atypical allergic phenotypes, particularly in endemic regions. These findings may have important implications for the diagnosis, management, and prevention of both anisakiosis and α-Gal syndrome and highlight the complex relationship between parasitic infection and food allergy pathogenesis. The findings support the hypothesis that *Anisakis* sp. infection may represent a novel route of sensitization to α-Gal. The work underscores the need to consider parasitic infections, particularly anisakiosis, as potential risk factors for atypical allergic phenotypes such as α-Gal syndrome, with important implications for clinical diagnosis and patient management. Further studies are warranted to clarify the clinical relevance of *Anisakis*-induced α-Gal sensitization, its potential role in α-Gal syndrome, and the mechanisms underlying the observed immunological cross-reactivity.

## Data Availability

The original contributions presented in this study are included in the article and in the Supplementary Materials. Further inquiries can be directed to the corresponding authors.

## Supplementary Materials

The following supporting information can be downloaded at: https://www.mdpi.com/article/10.3390/pathogens14080789/s1, Figure S1: Levels of anti-*Anisakis* sp. delipidated IgG (A), IgM (B), IgA (C), IgE (D), IgG4 (E), and IgE/IgG4 ratio (F) antibodies, expressed as optical density (O.D.), in patients diagnosed with gastroallergic anisakiosis (GAA), chronic urticaria with sensitization to *Anisakis* sp. (CU+), and chronic urticaria without sensitization to *Anisakis* sp. (CU−). AK+: GAA and CU+ patients; AK−: CU− patients; CU: UC+ and CU− patients; non-CU: GAA patients. Group comparisons were performed using Student’s *t*-test for normally distributed data and the Mann–Whitney *U* test for non-normally distributed data. * *p* < 0.05, ** *p* < 0.01, *** *p* < 0.001, **** *p* < 0.0001; Figure S2: Levels of anti-*Anisakis* sp. non-delipidated IgG (A), IgM (B), IgA (C), IgE (D), IgG4 (E), and IgE/IgG4 ratio (F) antibodies, expressed as optical densities (O.D.), in patients diagnosed with gastroallergic anisakiosis (GAA), chronic urticaria with sensitization to *Anisakis* sp. (CU+), and chronic urticaria without sensitization to *Anisakis* sp. (CU−. AK+: GAA and CU+ patients; AK−: CU− patients; CU: CU+ and CU− patients; non-CU: GAA patients. Group comparisons were performed using Student’s *t*-test for normally distributed data and the Mann–Whitney *U* test for non-normally distributed data. * *p* < 0.05, ** *p* < 0.01, *** *p* < 0.001, **** *p* < 0.0001; Figure S3: Levels of IgG (A), IgG4 (B), and IgE (C) antibodies against muscle tissue proteins from pork and beef, expressed as optical densities (O.D.), in patients diagnosed with gastroallergic anisakiosis (GAA), chronic urticaria with sensitization to *Anisakis* sp. (CU+), and chronic urticaria without sensitization to *Anisakis* sp. (CU−). AK+: GAA and CU+ patients; AK−: CU− patients; CU: CU+ and CU− patients; non-CU: GAA patients. Group comparisons were performed using Student’s *t*-test for normally distributed data and the Mann–Whitney *U* test for non-normally distributed data.
