# Supplementary material for: The Role of Anisakis sp. in α-Gal Sensitization: Implications for Parasitic-Induced Meat Allergy
Source: Pathogens. 2025 Aug 7;14(8):789. doi: 10.3390/pathogens14080789 (PMC12389079; doi:10.3390/pathogens14080789)
Supplement: Supplementary file 1 [file pathogens-14-00789-s001.zip › pathogens-3739580-supplementary.pdf]

Supplementary Materials

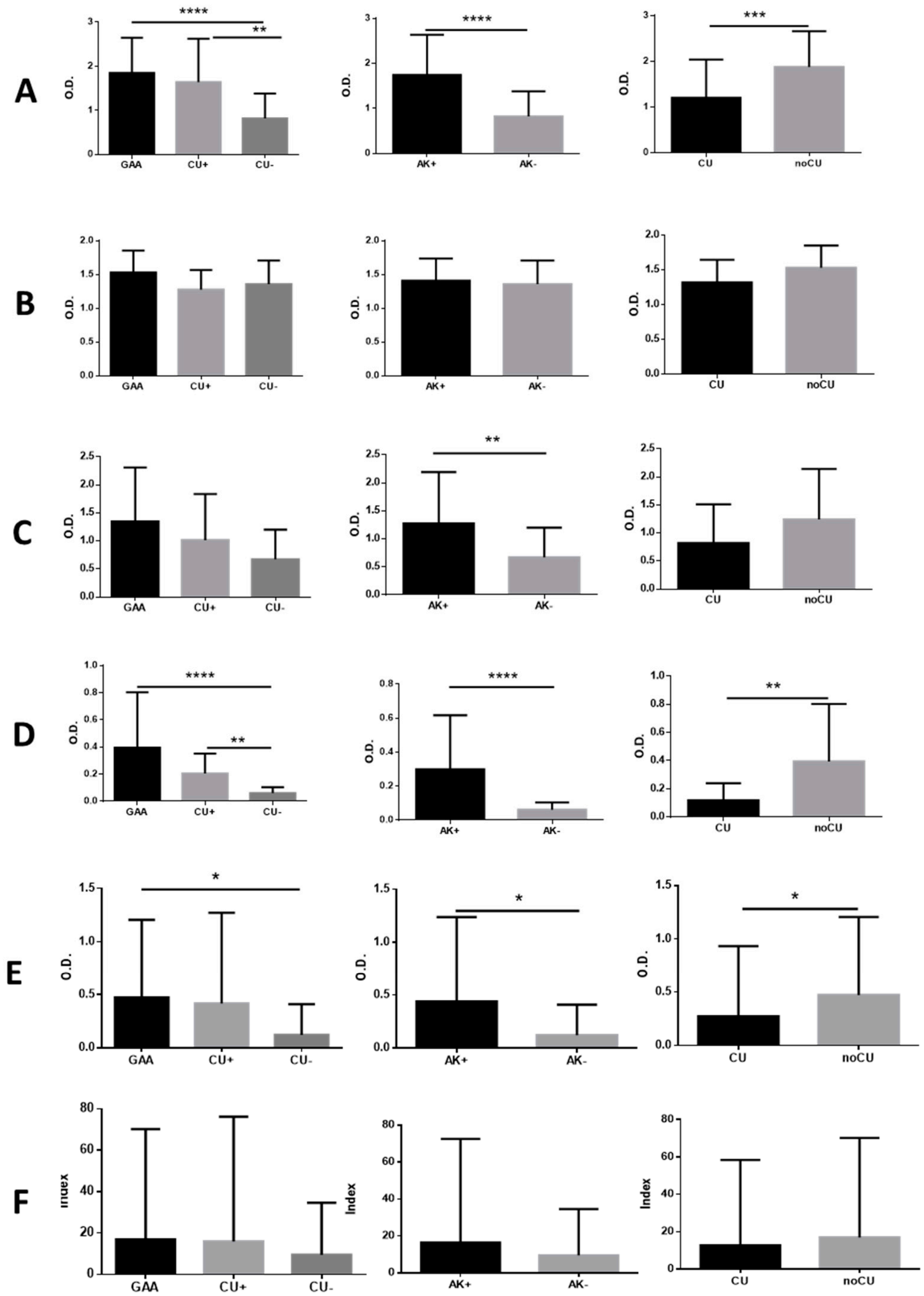

Figure S1. Levels of anti-*Anisakis* sp. delipidated IgG (**A**), IgM (**B**), IgA (**C**), IgE (**D**), IgG4 (**E**), and IgE/IgG4 ratio (**F**) antibodies, expressed as optical density (O.D.), in patients diagnosed with gastroallergic anisakiosis (GAA), chronic urticaria with sensitization to *Anisakis* sp. (CU+), and chronic urticaria without sensitization to *Anisakis* sp. (CU-). AK+: GAA and CU+ patients; AK-: CU- patients; CU: UC+ and CU- patients; non-CU: GAA patients. Group comparisons were performed using Student's *t*-test for normally distributed data and the Mann-Whitney *U* test for non-normally distributed data. *$p < 0.05$, **$p < 0.01$, ***$p < 0.001$, ****$p < 0.0001$.

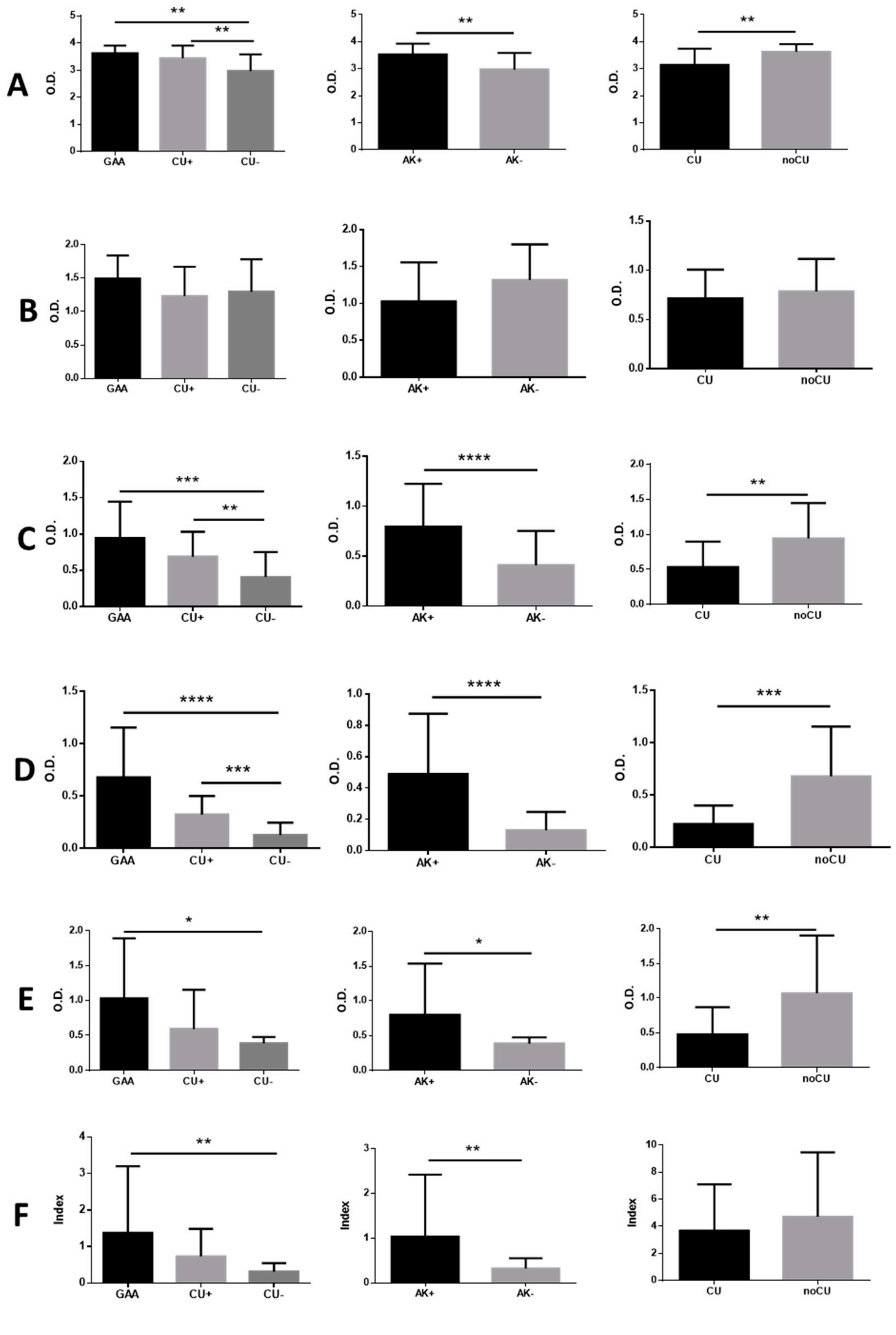

Figure S2. Levels of anti-*Anisakis* sp. non-delipidated IgG (**A**), IgM (**B**), IgA (**C**), IgE (**D**), IgG4 (**E**), and IgE/IgG4 ratio (**F**) antibodies, expressed as optical densities (O.D.), in patients diagnosed with gastroallergic anisakiosis (GAA), chronic urticaria with sensitization to *Anisakis* sp. (CU+), and chronic urticaria without sensitization to *Anisakis* sp. (CU-). AK+: GAA and CU+ patients; AK-: CU- patients; CU: CU+ and CU- patients; non-CU: GAA patients. Group comparisons were performed using Student's *t*-test for normally distributed data and the Mann-Whitney *U* test for non-normally distributed data. *$p < 0.05$, **$p < 0.01$, ***$p < 0.001$, ***$p < 0.0001$.

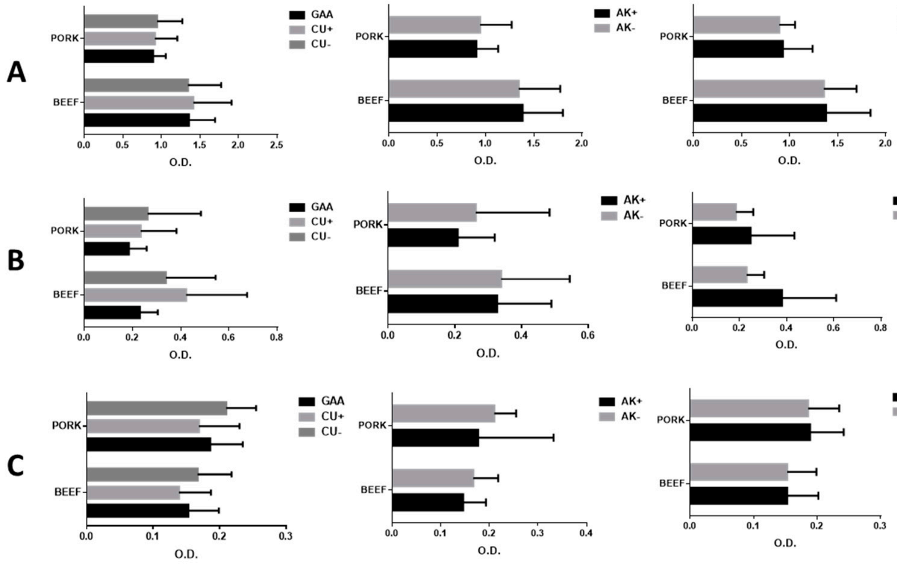

Figure S3. Levels of IgG (**A**), IgG4 (**B**), and IgE (**C**) antibodies against muscle tissue proteins from pork and beef, expressed as optical densities (O.D.), in patients diagnosed with gastroallergic anisakiosis (GAA), chronic urticaria with sensitization to *Anisakis* sp. (CU+), and chronic urticaria without sensitization to *Anisakis* sp. (CU-). AK+: GAA and CU+ patients; AK-: CU- patients; CU: CU+ and CU- patients; non-CU: GAA patients. Group comparisons were performed using Student's *t*-test for normally distributed data and the Mann-Whitney *U* test for non-normally distributed data.
